# Synthetic Biology: Applying Engineering to Life Sciences to Develop Rationally Designed Biological Parts, Devices, and Systems

**DOI:** 10.3389/fbioe.2013.00008

**Published:** 2013-10-09

**Authors:** Ianis G. Matsoukas

**Affiliations:** ^1^Engineering, Sports and Sciences Academic Group, The University of Bolton, Bolton, UK

**Keywords:** synthetic biology, DNA synthesis, bioinformatics and computational biology, pathway engineering, biofuels, therapeutics, artificial cells, artificial genome

Synthetic biology (SB) has attracted considerable attention in recent years. The term “SB” can be traced back to 1912 to Stéphane Leduc’s book *La Biologie Synthétique*. The next direct reference to the term is found in 1974 when Waclaw Szybalski, in only a few sentences, laid the foundations for the emerging field of SB. The new wave of SB aims to design and engineer biologically based parts, novel devices, and networks as well as redesigning existing, natural biological pathways. Hence, SB has the potential to deliver significant technological benefits and economic growth.

Synthetic biology has already made a resounding impact in fundamental and applied research. Currently it has broad applications in a number of areas including medicine, the environment and energy, agriculture, and food industries. Selected milestones and significant achievements include the reconstruction of a complete microbial genome (Gibson et al., 2010), the synthesis of a yeast chromosome (Dymond et al., 2011), and most recently, the engineering of *Saccharomyces cerevisiae* to produce high levels of a key precursor of the antimalarial drug artemisinin (Paddon et al., 2013).

*Synthetic Biology: Tools and Applications*, edited by Huimin Zhao (University of Illinois) provides a comprehensive coverage and a succinct overview of the state-of-the-art knowledge on SB (Zhao, 2013). The overall scope of the book is based on a review article (Zhao, 2013) published in *Wiley Interdisciplinary Reviews: Systems Biology and Medicine* (Liang et al., 2011). On more than 300 pages, structured into 17 chapters, *Synthetic Biology: Tools and Applications* brings together thought leaders and leading researchers in SB, by providing a systematic and integrated framework to examine key enabling components in SB.

The era of SB has arrived. As tellingly described in the introduction by the editor, SB builds on the advances in molecular, and systems biology and seeks to revolutionize life sciences in the same way that chemical synthesis revolutionized chemistry and integrated circuit design revolutionized computing. In addition, the number of publications on SB has increased exponentially in recent years. As a consequence, it is almost impossible to follow the rush of SB-literature in detail. Furthermore, the emergence of SB has been accompanied by the development of new and improved experimental tools and approaches. However, more exciting applications in basic and applied research would be useful to make SB easier, safer, and more predictable. Therefore, it is sound and timely to collect and converge the various research tools, methodologies, and prospects of SB research in a single comprehensive book.

The organization of the book into four well-chosen sections (rather than into 17 imposing chapters) lures the reader into reading a thematic section of several chapters at any one time, rather than the entire book. The order of topics is intended to represent a logical progression. Each chapter starts with an essential introduction; this allows reading the chapters independently from each other, a feature that readers will highly acknowledge.

The first thematic section, entitled “Synthesis and Engineering Tools in SB.” This section (chapters 1–4) addresses the latest state-of-the-art experimental tools and approaches developed for engineering biological systems at molecular, cellular, and multi-cellular levels. In addition, this section discusses the technologies for DNA synthesis and their limitations, whereas it also provides an overview of the key methods for protein engineering. The section closes by describing the challenges and opportunities in the standardization and modularization of biological parts, which is one of the key distinguishing features of SB. The second thematic section (chapters 5–8) reviews the mathematical modeling approaches, and *in silico* tools, which can dramatically accelerate the design process, as well as reduce the cost of development of novel devices and networks. In addition, this section introduces a new computational SB tool, the Synthetic Biology Software Suite, whereas a wide variety of successful examples are also discussed to highlight the effectiveness of these computational tools and approaches. The next section (chapters 9–13) deals with exciting practical applications of SB. Chapters of this section provide an overview on the potential therapeutic applications of SB on human health and disease, and on microbial production of pharmaceuticals. In addition, this section discusses the industrial application of SB in the microbial production of biofuels, and the challenges associated with biofuels production on an industrial scale. Furthermore, the available technologies and strategies for designing and constructing bacterial genomes, and the recent efforts on the analysis and creation of microbial consortia are also overviewed elegantly. The final section (chapters 14–17), Future Prospects, describes the current status of designing and constructing synthetic or artificial cells. This section also provides a comprehensive overview of the recent advances in cell-free SB. Furthermore, it provides an overview on the use of several tools to engineer light-energy conversion and carbon fixation in diverse non-photosynthetic hosts. Moreover, the recent developments and future prospects in engineering synthetic microbial ecosystems are also described. Finally, although an index is present, a glossary would be desirable.

*Synthetic Biology: Tools and Applications* uses a combination of elegant tables, figures, and graphs to present complicated information in a way that is accessible and understandable by the reader. All chapters are skillfully reviewed and highly citable. In summary, the book is an excellent addition to the current literature on SB. It is a perfect companion for policy makers and funding agencies. Lastly, but most importantly, *Synthetic Biology: Tools and Applications* will be greatly appreciated by geneticists, molecular biologists, bioinformaticians, physicists, chemists, and bioengineers, and those contemplating a future in SB.
